# Facial cutaneo-mucosal venous malformations can develop independently of mutation of *TEK *gene ﻿but may be associated with excessive expression of Src and p-Src

**DOI:** 10.1186/s12952-017-0072-5

**Published:** 2017-03-20

**Authors:** Nabila Brahami, Selvakumar Subramaniam, Moudjahed Saleh Al-Ddafari, Cecile Elkaim, Pierre-Olivier Harmand, Badr-Eddine Sari, Gérard Lefranc, Mourad Aribi

**Affiliations:** 1 Laboratory of Applied Molecular Biology and Immunology, University of Tlemcen, Imama-Mansourah, Rocade # 2, PO Box: 262, Tlemcen, 13000 Algeria; 2 UMR U866 INSERM, University of Burgundy, 21000 Dijon, France; 3 Laboratory of Cell and Hormonal Biology, Arnaud de Villeneuve Hospital, 34295 Montpellier, France; 4 Stomatology and Oral Surgery Department of Tlemcen, University Medical Centre, 13000 Tlemcen, Algeria; 5 Laboratoire d’Immunogénétique Moléculaire, Institut de Génétique Humaine, CNRS UPR 1142, et Université de Montpellier, Montpellier, 34095, Cedex 5 France

**Keywords:** Cutaneo-mucosal venous malformations, Direct sequencing, Germline and somatic DNA, p-Src, Src, *TEK* gene

## Abstract

We aimed to search for mutations in the germline and somatic DNA of the *TEK* gene and to analyze the expression level of Src and phospho-Src (p-Src) in tumor and healthy tissues from patients with facial cutaneo-mucosal venous malformations (VMCM). Eligible patients from twelve families and thirty healthy controls were recruited respectively at the Departments of Stomatology and Oral Surgery, and Transfusion Medicine of Tlemcen University Medical Centre. Immunoblot analyses of Src and p-Src were performed after direct DNA sequencing. No somatic or germline mutations were found in all the 23 exons and their 5’ and 3’ intronic flanking regions, except for one case in which a c.3025+20-3025+22 del mutation was highlighted at the intron 15, both in the germline and somatic DNA. Additionally, elevated expression levels of Src and p-Src were observed only in the patient with such mutation. However, when normalized to β-actin, the overall relative expression levels of both Src and p-Src were significantly increased in VMCM tissues when compared to healthy tissues (for both comparisons, *p* <0.001). In conclusion, we confirm the outcomes of our previous work suggesting that VMCM can develop independently of mutation of the *TEK* gene. Additionally, the results for Src activity are of particular interest in the context of specific targeted therapies and biological diagnosis. Nevertheless, such a conclusion should be confirmed through a mechanistic study and/or in a satisfactory number of patients.

## Background

Vascular malformations arise from an error of vascular morphogenesis and are named by their predominant vessel type: arterial, venous, capillary, lymphatic or different combinations of each of them [1]. Venous malformations (VMs) are the most frequent vascular abnormalities but remain quite rare, with an incidence of approximately 1 in 10,000 [2, 3]. They are present at birth, and often become apparent afterward. Rapid growth may occur during puberty, pregnancy, or traumatic injury [1].

When venous lesions are located both at skin and mucous membranes, VMs are called cutaneo-mucosal venous malformations (VMCMs). Their pathogenesis is not yet fully understood. Nevertheless, it is assumed to be caused by abnormal development of the venous system [4]. Further studies showed that somatic mutations in the gene of the receptor tyrosine kinase (*TEK/TIE2*, vascular endothelial cell specific receptor tyrosine kinase) was present in various single or multiple VMs and led to loss of TIE2 receptor function [5], and upregulated expression of other vascular endothelial growth factors, such as transforming growth factor (TGF)-β and fibroblast growth factor (FGF)-β, which exacerbated the severity of the lesion [6].

The TEK/TIE2 receptor tyrosine kinase plays a crucial role in angiogenesis and cardiovascular development [7]. The main role of this receptor is triggering angiogenesis signals leading to the formation of blood vessels. This signaling process facilitates communication between two types of cells within the walls of blood vessels, endothelial cells and smooth muscle cells [8]. Communication between these two cell types is necessary to direct angiogenesis and ensure the structure and integrity of blood vessels [9].

Angiogenesis, i.e. the formation of new blood vessels from preexisting ones, is a key event in tumor progression, which is controlled by a balance between positive and negative regulators [10, 11]. Among the several growth factors that can promote angiogenesis, vascular endothelial growth factor (VEGF) is the most widely studied and potent inducer of angiogenesis [12]. One group of signaling molecules that may be involved in the VEGF signaling cascade is the proto-oncogene tyrosine-protein kinase Src.

It has been reported that Src kinases play an important role in cell cycle control and cell adhesion and movement, as well as in cell proliferation and differentiation in a numerous cells and tissues [13]. They also play an important role in lymphokine-mediated cell survival and VEGF-induced angiogenesis [14]. Of note, Src protein is one of the best characterized non-receptor protein tyrosine kinases that are involved in receptor signaling and cell communication. Multiple cellular functions are attributed to the activity of Src as a molecular switch allowing the external signal transduction across the plasma membrane, and then its conversion into internal message upon activation of the target molecules inside a cell. High expression of Src has been reported to be associated with increased VEGF expression [15], cellular proliferation and angiogenesis [16].

On the basis of these reports, we extend previously published research on germline DNA of the *TEK* gene [17] by including new eligible patients with VMCMs and additional controls for the examination of both germline and somatic mutation, as well as the evaluation of Src and p-Src expression levels.

## Methods

### Study design

**Fig. 1:**
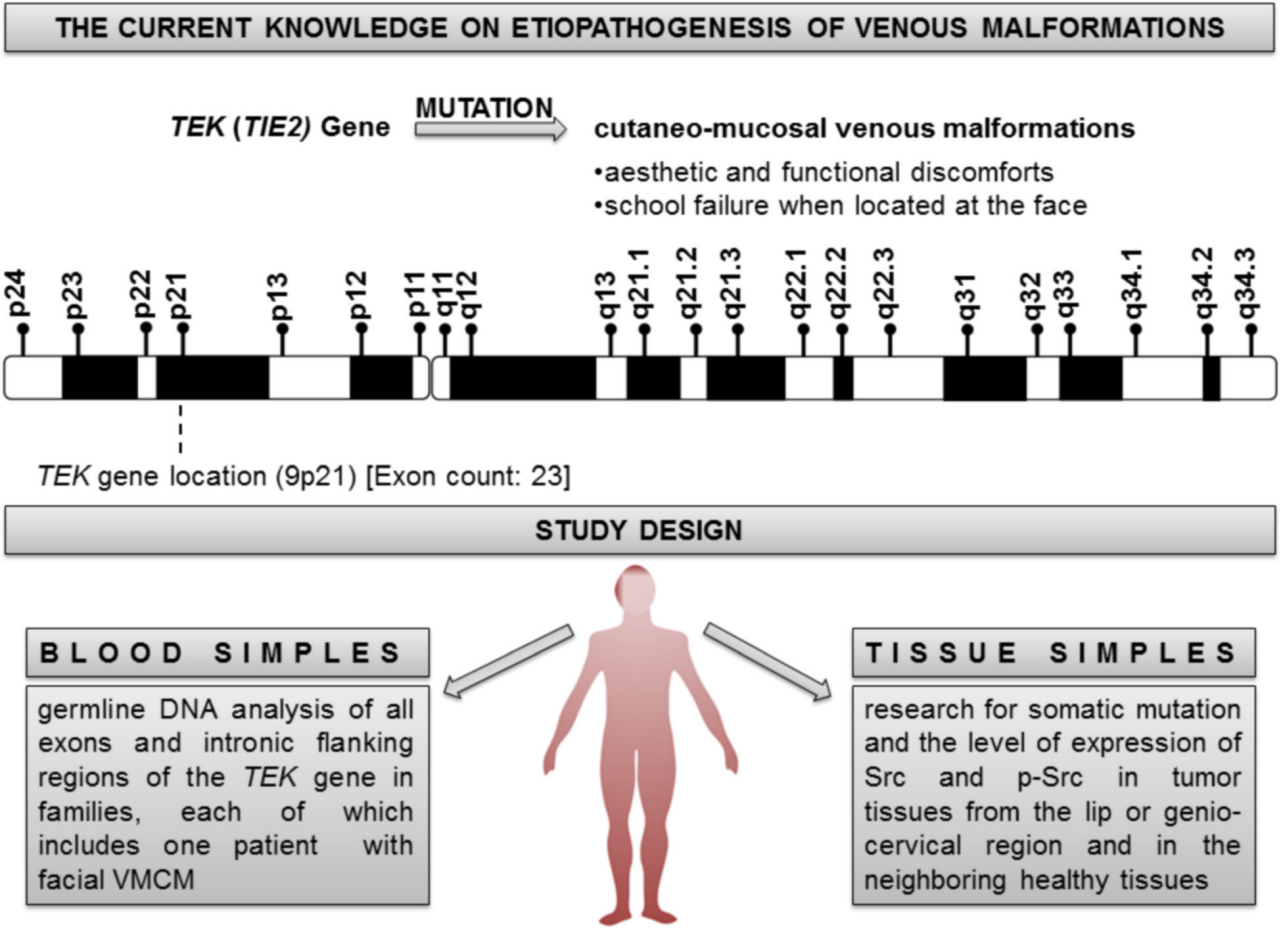
Study flow-chart. *TEK*: TEK tyrosine kinase endothelial (also known as *TIE2*), *TIE2*: tyrosine kinase with immunoglobulin and epidermal growth factor homology domains-2, VMCM: cutaneo-mucosal venous malformation

The study was performed in patients with VMCMs. The search for germline mutations in the DNA of *TEK* gene was carried out in patients and healthy controls. The search of somatic mutations and assessment of the expression of Src activity were performed in tumor and healthy tissues (Fig. 1).

### Patients and subjects

Recently, we carried out the germline DNA analysis of all exons of the *TEK* gene in ten families, each of them includes one patient with facial VMCM [17]. In addition to the ten patients, two new eligible cases and thirty matched healthy control volunteers were recruited respectively at the Departments of Stomatology and Oral Surgery, and Transfusion Medicine of Tlemcen University Medical Centre. The mean age (± standard error) of the patients (4 men and 8 women) at diagnosis was 13 ± 2 years (Table 1). The inclusion criteria were geographic location (North West of Algeria), and VMCM of the facial region. Patients were excluded in case of arteriovenous malformations. Patient recruitment was based on clinical history and examination. Superficial VMCM were diagnosed for the presence of a blue or purple depressible mass or under-mucous sheath, non-pounding and non-blowing yet increasing of volume when the patient is in an inclined position. The tumor mass tends to increase in size with effort and maneuvers that could increase pressure in the venous system. The histopathology examination was carried out after surgery. A magnetic resonance imaging (MRI) was performed to define the flow characteristics and the extension of the tumor.

**Table 1:** The demographic data of patients with cutaneo-mucosal venous malformations

Variable	Patients with VMCM
Age at diagnosis (year)	13 ± 2
Gender (F/M)	8/4
Total number of lesion (n)	1 ± 0
Lip VMCM (%)	11 (91.7)
Genio-cervical VMCM (%)	1 (8.3)

*VMCM* cutaneo-mucosal venous malformations

### Samples

Blood samples were collected into ethylenediaminetetraacetic acid-containing *Vacutainer* tubes (BD *Vacutainer* EDTA, USA). VMCM and normal tissues were taken from patients after surgery, immediately placed into a sterile collection tube in liquid nitrogen and, then, stored at – 80 °C in dry ice. Extracted DNA from blood samples and tissues were used for polymerase chain reaction (PCR) and direct DNA sequencing for all exons and their flanking regions of the *TEK* gene. Immunoblot analysis of Src, p-Src and β-actin expression were performed on tissues.

### DNA analysis

DNA extraction and purification was carried out as we described [17]. The search for mutation was performed by PCR amplification followed by direct sequencing of amplified DNA segments. Such analyses were performed in the Laboratory of Cell and Hormonal Biology, Arnaud de Villeneuve Hospital, Montpellier (France).

The primer sequences were specifically established to amplify each exon, using the Primer3 program v.0.4.0 [18], referring to the *TEK* gene sequence (ENSG00000120156) published in Ensembl [19] (Table 2).

**Table 2:** Sequences of the sense and antisense primers used for direct sequencing of all the exons of the *TEK* gene

Exon number	Sense and anti-sense primers 5’-3’
5’ UTR region	SP 5’-AGTCTGAGAAGGATTGGTCATCA-3’
	ASP 5’-CTGTCTGAGCACAGGGAGTTT-3’
Exon 1.2	SP 5’-CAGCCCTGCTGATACCAAAT-3’
	ASP 5’-CACTGATGAGATTTGGGGAGA-3’
Exon 2	SP 5’-GTTTACCCAATGGGGTCATG-3’
	ASP 5’-AGCAGCTGCCAAGACAAAAG-3’
Exon 3	SP 5’-AACGCATTAGCCACCACTGT-3’
	ASP 5’-ACATCTGCCCACAAGACCA-3’
Exon 4	SP 5’-CTGAATAGTTCAGCATTTTCATTCT-3’
	ASP 5’-CAATGCCTGGTTTTTGCTTA-3’
Exon 5	SP 5’-CTCCTTGTCTTTGTTTCTGTCG-3’
	ASP 5’-AAATTCTAGATCCAGCAACGATG-3’
Exon 6	SP 5’-GTTCATCCTACCATGCCACA-3’
	ASP 5’-TGATTCAAAATCCTGTTGTCCA-3’
Exon 7	SP 5’-AGTTGGCATGATAGGAGCTCA-3’
	ASP 5’-GGATGGAAACAAAAGAGGCTT
Exon 8	SP 5’-TCATCCACATCACAGGTGTCT-3’
	ASP 5’-GTCAGTTCTGCCTCTCCAGG-3’
Exon 9	SP 5’-TGGGGTCAATGTTATGGACC-3’
	ASP 5’-TCCTGGAAATTACCCCAAAG-3’
Exon 10	SP 5’-ATCACAAAACCTCAAAGCCG-3’
	ASP 5’-AGCCACCACCTTGAGGTAGA-3’
Exon 11	SP 5’-TTTCAAAAGCCTAATTTTCCTCA-3’
	ASP 5’-CACCCATTCAAAAGCGAACT-3’
Exon 12.1	SP 5’-AGTTGGCATGATAGGAGCTCA-3’
	ASP 5’-GGATGGAAACAAAAGAGGCTT-3’
Exon 12.2	SP 5’-TGGGGTCAATGTTATGGACC-3’
	ASP 5’-TCCTGGAAATTACCCCAAAG-3’
Exon 13	SP 5’-GCATAATGATCTAGGCCATGG-3’
	ASP 5’-CCTATAGGGCTGCACGGTAA-3’
Exon 14	SP 5’-GCTGCTGTTAAGTTCCCATTACA-3’
	ASP 5’-AAGCCAAAGAGAAGATGAGGC-3’
Exon 15	SP 5’-GTTCATCCTACCATGCCACA-3’
	ASP 5’-TGATTCAAAATCCTGTTGTCCA-3’
Exon 16	SP 5’-TTTGGTTGTATACAGTTGATGGTGA-3’
	ASP 5’-AGGCAAACCACAGCACAGTC-3’
Exon 17	SP 5’-GTTTACCCAATGGGGTCATG-3’
	ASP 5’-AGCAGCTGCCAAGACAAAAG-3’
Exon 18	SP 5’-TCTTCTGCCAAGATGTGGTG-3’
	ASP 5’-CAGGGGAGTACCTCGGAAA-3’
Exon 19	SP 5’-CTACCCAGCAATCATTTGTGG-3’
	ASP 5’-TGCTAATTTATTTCCTGAGCTTTTT-3’
Exon 20	SP 5’-GTGCAAGGGCCTATCCTAGG-3’
	ASP 5’-CCAAGTCACATCTGGTAGAACCA-3’
Exon 21	SP 5’-ATGTGCAGTGAGTTTGCCAA-3’
	ASP 5’-CGGCTGACTTTGCTAGAGTC-3’
Exon22	SP 5’-GTTTACCCAATGGGGTCATG-3’
	ASP 5’-AGCAGCTGCCAAGACAAAAG-3’
Exon 23.1	SP 5’-AGGTGGAATCAAAGCAGCCT-3’
	ASP 5’-CACGCCTTCCTATGAAGTCC-3’
Exon 23.2	SP 5’-AATCAGAATGCCTGTTTGTGG-3’
	ASP 5’-TTCTTAGGCTTGTAAGCAATGAG-3’
3’ UTR region	SP 5’-TCTCAATTTTATCCCTCACCTG-3’
	ASP 5’-TAAAGTATAATAAGGACATGTGGCA-3’

*SP* sense primer, *ASP* anti-sense primer, *UTR* untranslated region

The DNA was amplified in a thermocycler for PCR (Applied Biosystems, Foster, CA), using the primers described in Table 2. The medium of the DNA amplification reaction was composed of 50 ng of DNA, 25 $\mu$M of each primer, and 2X Promega PCR Master Mix (Promega). The PCR conditions were as follows: 5 min at 95 °C followed by 35 cycles of 30 s of denaturation at 95 °C, primer annealing at 60 °C for 30 s, and elongation at 72 °C followed by one cycle at 72 °C for 10 min.

After checking the quality and size of the PCR products by agarose gel (1.5%) electrophoresis, a bidirectional sequencing was performed by the use of Mix BigDye Terminator kit version 3.1 (ABI). The sequences of the 23 exons and their flanking regions were compared with the *TEK* gene reference sequence published in Ensembl using the SeqScape v2.5 software (ABI).

### Src, p-Src and β-actin immunoblot assays

Venous malformation and healthy control tissues were homogenised for 10 min each in lysis buffer (20 mM HEPES, pH 7.3; 1 mM EDTA; 1 mM EGTA; 0.15 mM NaCl; 1% Triton X-100; 10% glycerol; 1 mM phenylmethylsulfonyl fluoride; 2 mM sodium orthovanadate and 2 μl/ml anti-protease cocktail) and centrifuged (13000 *g* x 10 min). Protein concentrations in the supernatants were determined by bicinchoninic acid method (Pierce). Denatured proteins (40 μg) were separated by SDS-PAGE (10%) and transferred to PVDF membranes. Immunodetection was performed by using p-Src (cell signaling tech, OZYME, FRANCE), Src (cell signaling tech, OZYME, FRANCE) and β-actin (Sigma Aldrich, FRANCE) antibodies. β-actin was used as a loading control. Optimal dilutions of primary antibodies, including a monoclonal anti-β-actin, were 1:1000 (v/v). The horseradish peroxidase conjugated secondary antibodies were used at 1:5000 (v/v) dilution and the Enhanced Chemiluminescence (ECL) system (NEL121001EA, Perkin Elmer) was used for detection. Signal detection was done by ChemiDoc XRS System (Bio-Rad). Densitometry and protein band analysis were performed using ImageJ software (NIH, USA) as reported [20]. Such analyses were performed at the UMR U866 INSERM/Université de Bourgogne/AgroSup (France). Additional verification analyses and experiments were carried out at the Laboratory of Applied Molecular Biology and Immunology (University of Tlemcen, Algeria).

## Results and discussion

Facial VMCMs are often responsible for aesthetic and functional discomfort, but also cause detrimental changes in personal relationships, especially during childhood and adolescence. They are due to localized defects of angiogenesis that are caused by genetic modifications and anomalies in signaling pathways, including that of Src family kinases. From a genetic point of view, studies of rare familial cases have helped to suggest that these defects could be the result of mutations in the *TEK* gene (also referred to as *TIE2*), which is located on the band 21 of the short arm of chromosome 9 (9p21).

It has been reported that *TEK* is the only gene which mutations that can cause the development of VMCMs [21]. As a matter of fact, the *TEK* gene was originally identified as a factor responsible for these defects thanks to a linkage analysis conducted in some families with autosomal dominant transmission [4, 22]. Mutated gene isolated by positional cloning experiment and the use of proteins expressed in insect cells have demonstrated that the mutation results in increased activity of the receptor tyrosine kinase TIE2, i.e. the angiopoietin receptor which is known to be specific for vascular endothelial cells. This mutation corresponds to a missense mutation resulting in an arginine-to-tryptophan substitution at position 849 (R849W) in the kinase domain of TIE2 [4].

**Fig. 2:**
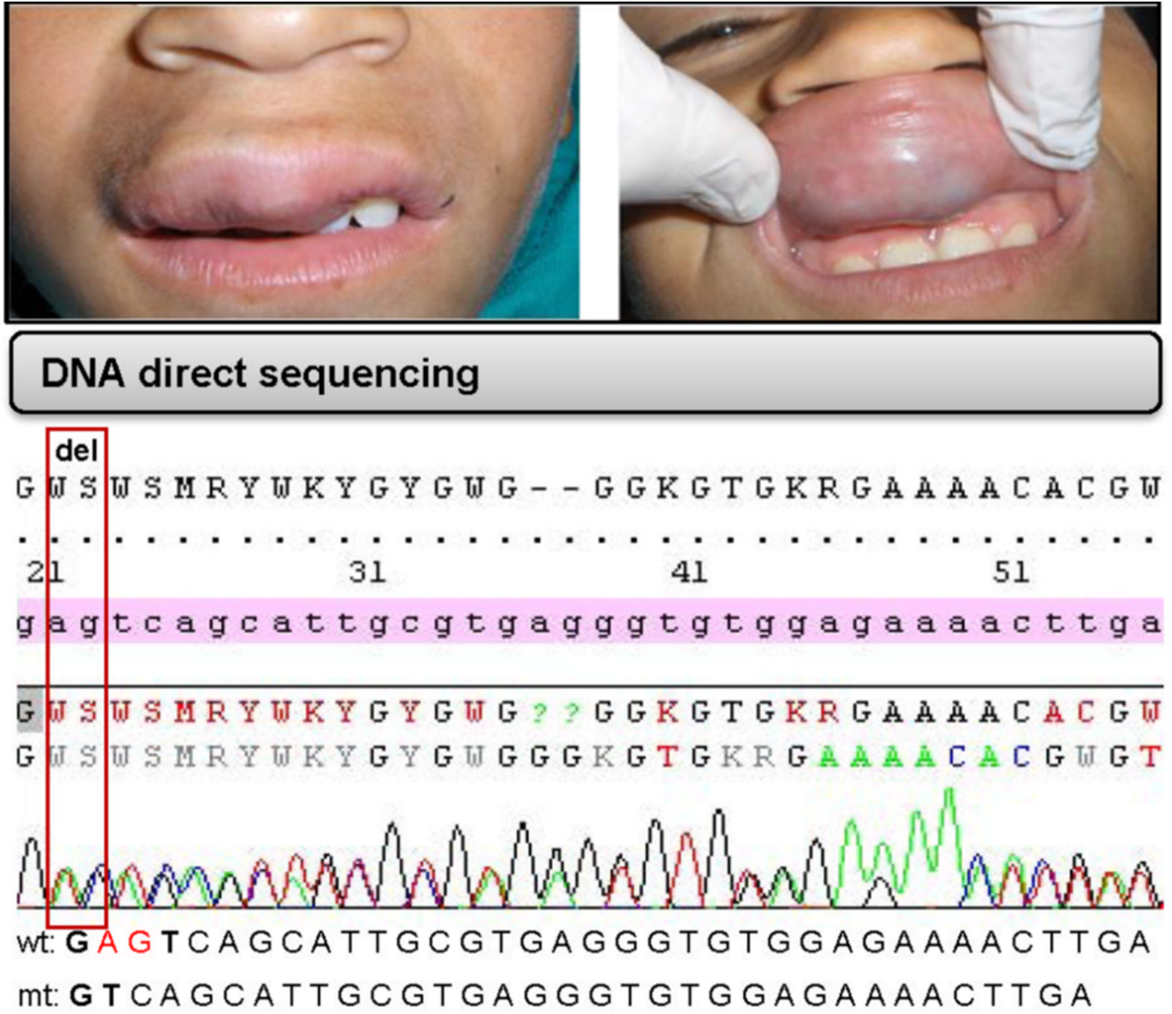
Localization of venous malformations on mucosal sides of the uper lip and results of direct sequencing of a part of intron 15 in germline and somatic DNA of the *TEK* gene. The patient with the malformation was diagnosed at the age of 11 years. No same cases have been identified in its first degree family. The representative electropherogram of the same *TEK* frameshift mutation (c.3025+20-3025+22 del) detected at the germline and somatic DNA level indicates a deletion of two nucleotides at the intron 15. The red box indicates the position of such deletion. Wild-type and mutant *TEK* DNA sequences are shown along. mt: mutant, VMCM: cutaneo-mucosal venous malformations. wt: wild-type

It has previously been reported an in-frame deletion of 129-bp, which corresponds to a loss of exon 3 and part of exon 4, from a patient by cDNA screening [5]. In the current study, we focused our experiments on patients from the North-West region of Algeria, which is usually characterized by a particular socio-demographic context presenting a high rate of consanguineous marriage [3, 23, 24]. So it is well-established that consanguinity causes excessive homozygosity and loss of heterozygosity (LOH) [25]. However, the most common R849W-TIE2 substitution that induce in vitro ligand-independent hyperphosphorylation, occurred in 10 patients from 17 Belgium families reported by Limaye team [4, 21, 26], has been shown in the context of heterozygosity. For our part, we have recently shown, using a direct sequencing of all exons of germinal DNA, including 5’ and 3’ intronic flanking sequences, that VMCMs could develop in the absence of mutation in the *TEK* gene. In order to check our results and to obtain more extensive information, we examined somatic mutation and the expression levels of Src and p-Src in tumor and neighboring healthy tissues. So direct sequencing of the amplification products, from germinal and somatic DNA of the *TEK* gene, revealed no mutation in all the 23 exons and their 5’ and 3’ intronic flanking regions, except for one patient in which a deletion of two nucleotides intronic c.3025+20-3025+22 del was found at the intron 15, both in germline and somatic DNA (Fig. 2). The analysis of the consequences of this deletion on splicing intron of exon 15, by the program “Splice site analysis” in Human Splicing Finder v 2.4.1 [27] shows that there is no splice donor site creation and there is no splice acceptor of interest. Nevertheless, it has been reported that two unusual mutations that are not predicted by bioinformatics analysis to induce significant exon skipping, have been found to have an effect on pre-mRNA splicing [28]. Consequently, defects in pre-mRNA splicing may represent a cause of a change in TEK protein activity. Additionally, intronic mutations may lead to retention of large segments of intronic DNA, or to removing exons, which lead to the production of non-functional proteins. Other intronic variants can interfere with those that regulate genes expression, such as nonsense-mediated decay (NMD) [29] and export of mRNA from nucleus to the cytoplasm [30].

**Fig. 3:**
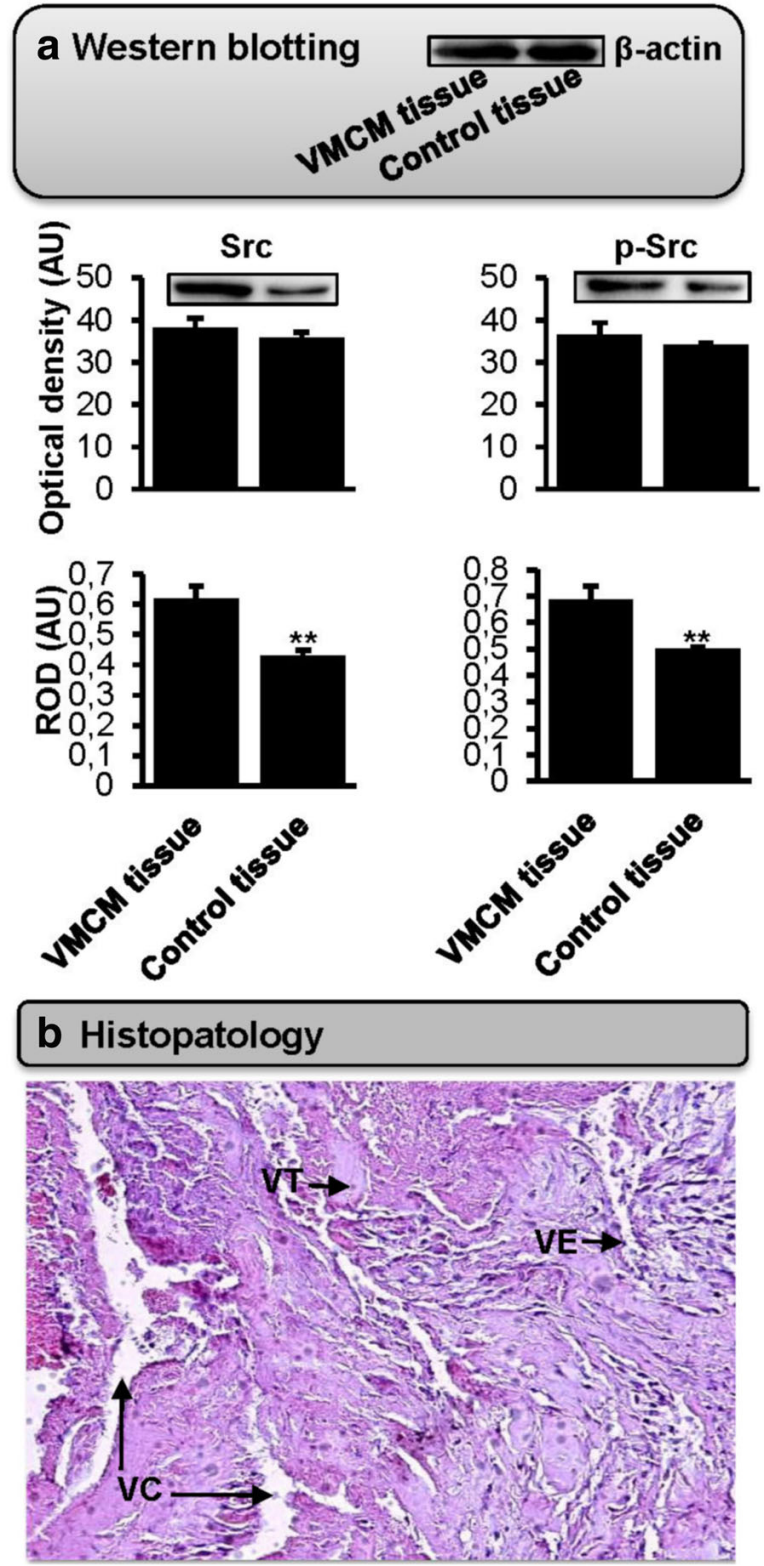
Expression of Src in facial venous malformation and associated histopathology features. **a** VMCM tissues from the lip or genio-cervical region and neighboring healthy control tissues (*n* = 12/12) were analyzed by western blotting for the expression of signaling molecules. Densitometry and protein band analysis were performed using ImageJ software (NIH, USA). The mean optical density values (in arbitrary units, AU) of the Western blotting bands are given in percentage related to the total area for each band ± standard error of mean. The relative expression of Src and p-Src were normalized to β-actin as a loading control. The image bands correspond to VMCM tissue versus healthy control tissue in the patient with the deletion of the two nucleotides “CT” in intron 15 of the *TEK* gene (the relative expression ratios between VMCM tissue versus healthy tissue were 2.3 for Src and 1.9 for p-Src). The statistical graphs represent the results of all VMCM and healthy control tissues. *P*-values for optical density and ROD were respectively greater than 0.05 and less than 0.001 for both Src and p-Src by Mann–Whitney *U* using SPSS software version 16.0 (SPSS Inc., Chicago, IL, USA). **b** Histological layers stained with hematoxylin-eosin showed thick and hyaline vessels with vascular thrombosis and bordered venous lakes with endothelial cells (H-E x 10). ROD: relative optical density, VC: vascular cavity, VE: vascular endothelium, VMCM: cutaneo-mucosal venous malformation, VT: vascular thrombosis

On the other hand, western blotting analyses showed an elevated expression of Src and p-Src only in the patient with such mutation. However, the overall relative expression levels of both Src and p-Src related to β-actin were significantly increased in VMCM tissues when compared to healthy tissues (for the two comparisons, *p* <0.001) (Fig. 3). Our finding would add new mechanistic information that should be very interesting in the diagnosis and treatment targeting angiogenesis, which is specifically engaged in the process of VMCM development.

Angiogenesis and blood vessel formation involves many signaling pathways that may interact with each other via Src [31, 32]. Src is considered as the focus of a variety of signaling pathways. It can be activated in multiple ways to become p-Src, which can in turn activate specific signaling pathways through phosphorylation of target proteins [33, 34]. In our study, the increased expression of Src and p-Src would be associated with the inducible effects of some angiogenic growth factors, including VEGF, but also the basic fibroblast growth factor (bFGF). Indeed, it has previously been reported that these two factors initiate the Src kinases signaling pathways, leading to the increased expression of Src in angiogenic tissues [14].

Although both VEGF and FGF stimulate Src activation in avian endothelial cells, only VEGF-induced angiogenesis is inhibited by treatment with a retrovirus that encodes for Src-251, which suppresses both angiogenesis and tumor growth. Moreover, overexpression of Src-251 in avian blood vessels induces apoptotic death, indicating that VEGF-induced activation of Src is essential for endothelial cell survival and angiogenesis. Similar results have been obtained in mice using a retrovirus encoding for the C-terminal Src kinase (CSK) a tyrosine kinase protein that blocks the action of Src through phosphorylation of the inhibitory site on Tyr527 [14].

The extended Src family includes at least ten proteins (Src, Frk, Lck, Lyn, Blk, Hck, Fyn, Yrk, Fgr, and Yes) [35] that engage jointly in the intracellular signal transduction [34, 36–38]. Numerous studies have shown an increase in Src and p-Src expression levels in tissues of different tumors, such as breast cancer, osteosarcoma and squamous cell carcinoma of the tongue [39–41]. Additionally, it has recently been shown that increased expression of Src is positively correlated with metastasis [42, 43].

A relationship between the *TEK* gene and Src signaling pathway can be suspected in the context of VEGF costimulation. Indeed, angiopoietin 1 (Ang1) activates TEK receptor, which triggers the activation of Rous sarcoma virus (Ras) homologous A (RhoA), which, in turn, inhibits Src proteins [44]. It has recently been reported that intact TIE2 may be necessary to blunt Src activation [45]. In our study, the dinucleotide deletion at intron 15 of the *TEK* gene may affect the function of this protein and consequently lead to an increased expression of Src and p-Src in VMCM tissue.

## Conclusions

Here we confirm that VMCMs, especially non-family VMCMs, are not necessarily linked to mutations in the *TEK* gene. Although increased relative expression of the Src protein appears to be associated with VMCMs, such outcomes deserve to be verified in various populations. Indeed, this is a novel report on relative issues and an alternative reference for biological diagnosis and specific targeted treatment of angiogenesis, using monoclonal antibodies or pharmacological inhibitors. In order to confirm the efficacy of this approach, further investigations should be conducted, and among others, it would be wise to conduct a mechanistic study researching the link with the Src pathway.

## Abbreviations

CSK: C-terminal Src kinase
DNA: Deoxyribonucleic acid
FGF: Fibroblast growth factor
MRI: Magnetic resonance imaging
PCR: Polymerase chain reaction
p-Src: Phosphorylatyed Src
R849W: Arginine-to-tryptophan substitution at position 849
Ras: Rous sarcoma virus
RhoA: Ras homologous A
Src: Proto-oncogene tyrosine-protein kinase
*TEK*: Vascular endothelial cell specific receptor tyrosine kinase (also referred to as *TIE2*)
TGF: Transforming growth factor
*TIE2*: Endothelial tyrosine kinase receptor
VEGF: Vascular endothelial growth factor
VM: Venous malformation
VMCM: Cutaneo-mucosal venous malformation
VPF: Vascular permeability factor
